# Latent tuberculosis infection in myasthenia gravis patients on immunosuppressive therapy: high incidence yet moderate reactivation rate

**DOI:** 10.1080/07853890.2023.2282182

**Published:** 2023-11-14

**Authors:** Kangzhi Chen, Fei Jiang, Qian Zhou, Xiaohua Dong, Ting He, Yi Li, Zhaohui Luo, Weiwei Duan, Huan Yang

**Affiliations:** Department of Neurology, Xiangya Hospital, Central South University, Changsha, China

**Keywords:** Myasthenia gravis, latent tuberculosis infection, immunosuppressive therapy, prevalence, active tuberculosis

## Abstract

**Background:**

Immunosuppressive therapies (ISTs) are mainstays for management of myasthenia gravis (MG). Meanwhile, latent tuberculosis infection (LTBI) is common in the setting of high-burden countries. However, the prevalence of LTBI among MG patients and whether receiving ISTs for MG would aggravate LTBI reactivation remain unknown.

**Methods:**

We retrospectively analyzed the frequency of LTBI *via* interferon-gamma release assay (IGRA) positivity among hospitalized MG patients from both rural and urban areas in a tertiary hospital, and those receiving ISTs were followed up to investigate the reactivation risk of LTBI.

**Results:**

A total of 300 MG patients with determinate IGRA results were enrolled, where the frequency of LTBI was 35.0%. Male (OR = 1.910, 95% CI: 1.181–3.089, *p* = .008) and elderly (OR = 1.044, 95% CI: 1.027–1.061, *p* < .001) patients were prone to LTBI. Of those with LTBI, 78 individuals on ISTs were successfully followed up for a median duration of 18.3 (8.5–24.0) months, of which 25 (32.1%) received anti-tuberculosis (TB) treatments. The rate of various degrees of adverse events was 82.1% over the course of the follow-up, but was not different between individuals with and without therapies against TB (*χ*^2^ < 0.001, *p* > .999). Only 1 patient eventually reported lymph node and intestinal TB, with the incidence rate of LTBI reactivation preliminarily estimated to be 0.81 per 100 person years.

**Conclusion:**

The frequency of LTBI is high in our MG cohort, especially among those with advanced age and males. However, receiving immunosuppressives seems not to increase the risk of LTBI reactivation. LTBI screening is strongly recommended for all MG patients ready to receive ISTs, while preventive anti-TB chemotherapy should be prescribed after weighing potential benefits against the risk of side effects in those with LTBI. In-depth investigation is still entailed to further verify these findings due to the limitation of the retrospective single-center design of our study.

## Introduction

Tuberculosis (TB) is a prevalent contagious disease caused by *Mycobacterium tuberculosis* (MTB) and represents one of the leading causes for ill conditions and deaths in the world with numerous newly-diagnosed and drug-resistant cases [1]. Generally, latent tuberculosis infection (LTBI) and active tuberculosis (ATB) are recognized as two major distinctive states of MTB infections, where LTBI refers to a condition with sustainingly poised immune responses primed by MTB antigens yet without either evident clinical manifestations or actively pathogenic replication of the agents [2]. Nevertheless, a fraction of LTBI may convert to ATB. The absolute risk of TB in all kinds of contacts was 1.7 per 100 person years for those with a positive interferon-gamma release assay (IGRA) result after a follow-up of at least 12 months, including those using immunosuppressants as high-risk groups [3]. China ranks the third country with a high burden of TB, accounting for 7.4% of the overall estimated cases globally [1]. Quite a few asymptomatic individuals may be positive for IGRA, and it is thus urgent to deal with the large populations potentially afflicted with TB, especially in patients complicating with other diseases.

Myasthenia gravis (MG) is a prototypical autoimmune disease characterized by autoantibodies against acetylcholine receptor (AChR), muscle-specific tyrosine kinase (MuSK) or other postsynaptic muscle-associated proteins, and immunosuppressive therapies (ISTs) are inevitable for managing MG [4]. In recent years, extensive investigations on the efficacy and optimal regimens of existing medications are ongoing [5–7]. Besides, a host of novel biologic agents have been also developed for treating MG [8]. However, the eligibility of these clinical trials in principle excludes MG patients combined with chronic or recurrent infections including LTBI. It therefore brings about a dilemma of whether and when MG patients with LTBI should receive ISTs. So far, no study has investigated the incidence rate as well as the reactivation rate of LTBI among MG patients. Herein, we preliminarily unraveled the prevalence of LTBI in our MG cohort, and deciphered the incidence rate of ATB in those with LTBI on ISTs.

## Methods

### Patients and data collection

Inpatients with MG in the Department of Neurology of Xiangya Hospital from January 2017 to September 2021 screened for LTBI around the hospitalization period were preliminarily included. MG diagnosis was based on fluctuant changes of myasthenic weakness supported by positive neostigmine test or clinical responses to acetylcholinesterase inhibitors, abnormal repetitive nerve stimulation tests, and/or seropositivity of autoantibodies to AChR or MuSK [4]. LTBI was defined as IGRA positivity without symptomatic, radiographic and/or microbiological evidence of ATB [2], while patients with manifestations mimicking ATB were evaluated by respiratory physicians to exclude the possibility of ATB. MG patients with LTBI on ISTs, including glucocorticoids (GCS), tacrolimus (TAC), mycophenolate mofetil (MMF), or azathioprine (AZA), for at least 3 months during the follow-up period, were included for further analysis. The dosage of GCS was converted to that of equivalent prednisone for consistency.

**Figure 1.**
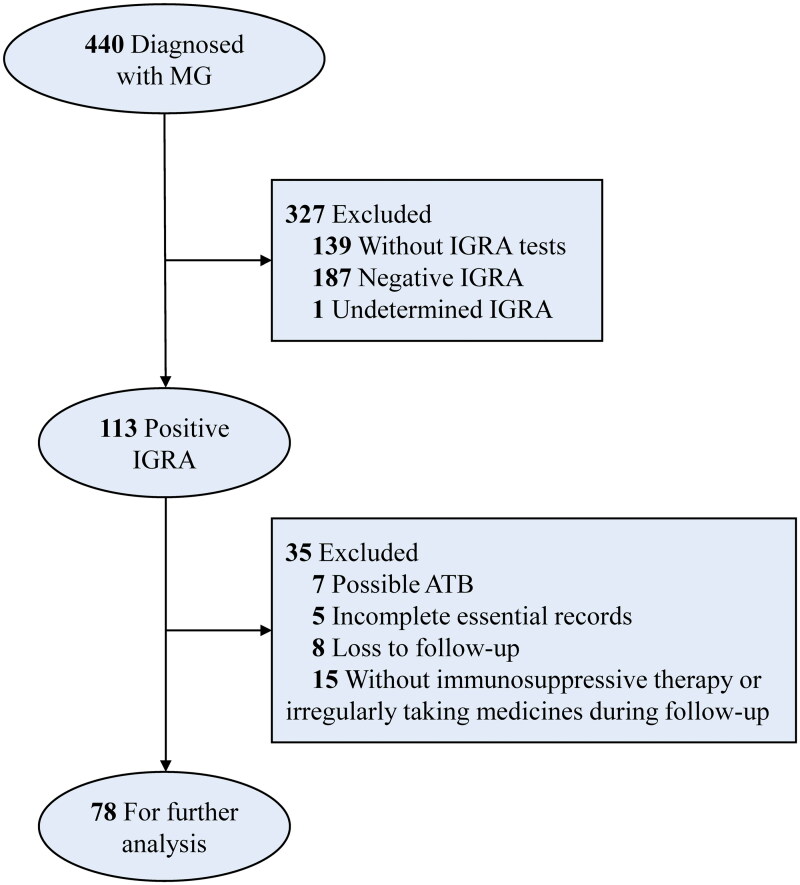
The flow chart on the process of including and excluding cases for specific reasons. ATB: active tuberculosis; IGRA: interferon-gamma release assay; MG: myasthenia gravis.

The observation endpoint was set at the last effective in-person visit to our hospital when maximum data associated with MG condition, TB status or adverse events (AEs), could be obtained, or the last follow-up *via* the structured telephone interview. The outcome of particular concern was the presence of symptoms/signs and examinations indicating LTBI reactivation. The ISTs and anti-TB therapy regimens, symptoms and signs of ATB, and AEs during the follow-up were either accessed *via* clinical records or reported by patients in the telephone interview. Details for inclusion and exclusion criteria are demonstrated in the flowchart (Figure 1).

### Statistical analysis

The description of continuous variables was presented as median (inter-quartile range, IQR), and categorial variables were described as frequency (percentage). The comparison of continuous and categorial variables was achieved *via* Mann-Whitney U test, or *via* Chi-squared test, adjusted Chi-squared test or Fisher’s exact test as appropriate, respectively. To analyze the effects of age and sex on IGRA positivity and LTBI frequency, a univariate Logistic regression model was applied. Wilcoxon signed ranks test was performed to compare variables at the baseline and the endpoint. The statistical analysis was conducted *via* IBM SPSS Statistics for Windows, version 26 (IBM Corp., Armonk, N.Y., USA). Part of the diagrams were visualized *via* package ‘ggplot2’ in R version 4.0.4 (R Foundation for Statistical Computing, Vienna, Austria).

## Results

### Incidence and distribution of MG patients with positive IGRA and LTBI

**Figure 2.**
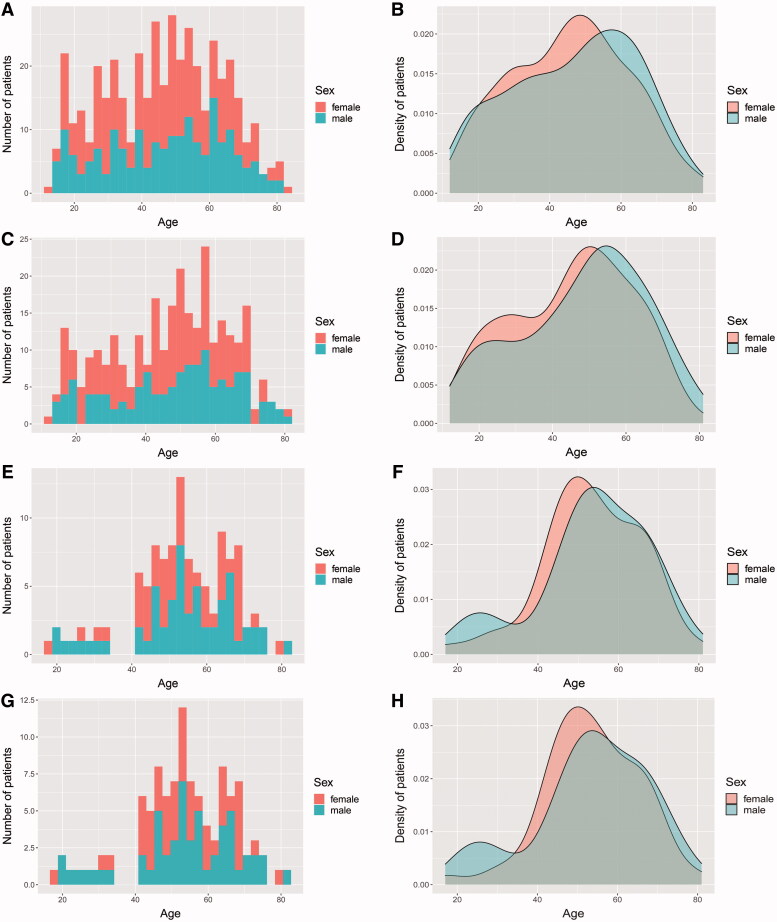
The demographic distributions of the whole populations with MG (A,B), individuals with determinate IGRA results (C,D), those with positive IGRA results (E,F), and those with LTBI (G,H). Both the number (A,C,E,G) and the density (B,D,F,H) of patients at different ages subdivided by sex are presented.

We included 440 MG patients in all for screening LTBI with a median age of 47 (32.25–59.75) years, of which 252 (57.3%) were females (Figure 2A,B). During or around the hospitalization, 113 were tested positive for IGRA among 300 patients with determined IGRA results. In the latter subgroup, 171 (57.0%) were females, with a median age of 49 (33–59) years (Figure 2C,D), which was in parallel in the sex ratio and age distribution with the whole population that weakened the selection bias. Taken separately, the frequency of positive IGRA in screened male MG patients was even higher (OR = 1.936, 95% CI: 1.205–3.110, *p* = .006). Those with advanced age were also inclined to be positive for IGRA (OR = 1.047, 95% CI: 1.030–1.064, *p* < .001).

Of 113 IGRA-positive patients, 46.9% were females, with a median age of 54 (47–64) years (Figure 2E,F). A total of 112 patients were evaluated *via* pulmonary radiographic examinations and 12 underwent microbiologic tests, of which 20 exhibited imaging features of pulmonary TB but none was sputum-positive. Specifically, one patient was diagnosed with tuberculous pleuritis, while 5 patients presented with pulmonary lesions potentially indicating ATB. Besides, another was afflicted with tubercular meningitis though no pulmonary lesion for TB was observed. After ruling out 7 cases of potential ATB and 1 case lacking effective evaluation, 105 patients were either clinically or microbiologically regarded as LTBI, thus with the incidence of LTBI being 35.0%. Of them, 46.7% were females, with a median age of 54 (47–64) years (Figure 2G,H). Likewise, the frequency of LTBI was higher in male (OR = 1.910, 95% CI: 1.181–3.089, *p* = .008) and elderly (OR = 1.044, 95% CI: 1.027–1.061, *p* < .001) MG patients among those with determinate IGRA results.

### Characteristics of MG patients with LTBI receiving ISTs

Following excluding the unqualified cases for further analysis (Figure 1), we deciphered baseline characteristics of 78 finally enrolled MG patients with LTBI receiving ISTs during the follow-up. Specifically, female patients exhibited a modest dominance (55.1%), and the median age of all patients was 53 (46.75–60) years old. The phenotype features of MG patients were detailed in Table 1. Of note, 41 patients (52.6%) were already exposed to ISTs at the enrollment. The biomedical features of enrolled MG patients with LTBI were also examined and shown in Table 2, partially reflecting the impacts of ISTs on immune responses such as the changes in the blood cell count and the level of immunoglobulins.

**Table 1.** Clinical features of patients with MG and LTBI receiving immunosuppressive therapy.

Variables	Total (*n* = 78)	With a history of TB or presence of pulmonary lesions (*n* = 15)	Without a history of TB and absence of pulmonary lesions (*n* = 63)	*p*-value
Sex ratio (female to male)	1.23 (43:35)	0.67 (6:9)	1.42 (37:26)	.190
Age (years old) [median (IQR)]	53 (47–60)	53 (45–65)	53 (47–60)	.643
Disease duration (m) [median (IQR)]	13.5 (4–81.5)	30 (5–108)	12 (4–71.5)	.477
Onset type				.521
Early-onset, *n* (%)	41 (52.6%)	9 (60%)	32 (50.8%)	
Late-onset, *n* (%)	37 (47.4%)	6 (40%)	31 (49.2%)	
MGFA classification nadir				.545
IIa, *n* (%)	5 (6.4%)	1 (6.7%)	4 (6.3%)	
IIb, *n* (%)	4 (5.1%)	1 (6.7%)	3 (4.8%)	
IIIa, *n* (%)	7 (9.0%)	2 (13.3%)	5 (7.9%)	
IIIb, *n* (%)	13 (16.7%)	2 (13.3%)	11 (17.5%)	
IVa, *n* (%)	7 (9.0%)	3 (20.0%)	4 (6.3%)	
IVb, *n* (%)	37 (47.4%)	5 (33.3%)	32 (50.8%)	
V, *n* (%)	5 (6.4%)	1 (6.7%)	4 (6.3%)	
Baseline QMG score [median (IQR)]	18 (14–22)	20 (15–22)	18 (14–22)	.457
Antibody profile				>.999
AChR-Ab, *n* (%)	61 (78.2%)	12 (80%)	49 (77.8%)	
MuSK-Ab, *n* (%)	7 (9.0%)	1 (6.7%)	6 (9.5%)	
Seronegative, *n* (%)	2 (2.6%)	0 (0%)	2 (3.2%)	
Unknown, *n* (%)	8 (10.3%)	2 (13.3%)	6 (9.5%)	
Thymoma, *n* (%)	28 (35.9%)	5 (33.3%)	23 (36.5%)	.818
Immunosuppressive therapy^a^				
Exposure ever, *n* (%)	41 (52.6%)	6 (40%)	35 (55.6%)	.278
Time since start (m) [median (IQR)]	15 (1.5–60)	43.5 (19.88–77)	13 (1.5–59)	.160
GCS				
Exposure ever, *n* (%)	36 (46.2%)	6 (40%)	30 (47.6%)	.595
Average daily dose (mg) [median (IQR)]	15.43 (5.77–25.00)	10.52 (4.78–22.35)	16.08 (7.27–25.66)	.396
TAC				
Exposure ever, *n* (%)	18 (23.1%)	4 (26.7%)	14 (22.2%)	.979
Average daily dose (mg) [median (IQR)]	0.87 (0.37–2.08)	0.39 (0.18–0.79)	1.58 (0.61–2.48)	.089
MMF				
Exposure ever, *n* (%)	8 (10.3%)	2 (13.3%)	6 (9.5%)	>.999
Average daily dose (g) [median (IQR)]	0.45 (0.14–0.73)	NA^b^	0.63 (0.28–0.76)	NA
AZA				
Exposure ever, *n* (%)	9 (11.5%)	2 (13.3%)	7 (11.1%)	>.999
Average daily dose (mg) [median (IQR)]	14.89 (7.70–56.11)	NA^b^	19.67 (11.32–85.94)	NA
PE^a^				
Exposure ever, *n* (%)	15 (19.2%)	3 (20%)	12 (19%)	>.999
Average session [median (IQR)]	2 (1–2)	NA^b^	2 (1–2)	NA
IVIG^a^				
Exposure ever, *n* (%)	7 (9.0%)	1 (6.7%)	6 (9.5%)	>.999
Average session [median (IQR)]	1 (1–3)	NA^b^	1 (1–2)	NA
Thymectomy, *n* (%)	18 (23.1%)	2 (13.3%)	16 (25.4%)	.512
Time since thymectomy (m) [median (IQR)]	18.8 (2.9–86.1)	NA^b^	18.8 (3.1–86.5)	NA
History of MC, *n* (%)	9 (11.5%)	2 (13.3%)	7 (11.1%)	>.999
Comorbidities				
Hypertension, *n* (%)	26 (33.3%)	5 (33.3%)	21 (33.3%)	>.999
Diabetes mellitus, *n* (%)	16 (20.5%)	2 (13.3%)	14 (22.2%)	.681
Other autoimmune diseases, *n* (%)	6 (7.7%)	3 (20%)	3 (4.8%)	.147
HBV infection, *n* (%)	8 (10.3%)	0 (0%)	8 (12.7%)	.325

AChR-Ab: acetylcholine receptor antibody; AZA: azathioprine; GCS: glucocorticoids; HBV: hepatitis B virus; IQR: inter-quartile range; IVIG: intravenous immunoglobulin; MC: myasthenic crisis; MGFA: Myasthenia Gravis Foundation of America; MMF: mycophenolate mofetil; MuSK-Ab: muscle-specific tyrosine kinase antibody; NA: not applicable; PE: plasma exchange; QMG: Quantitative Myasthenia Gravis; TAC: tacrolimus.

^a^ The median (IQR) of the average daily doses of immunosuppressives and the average sessions of PE and IVIG was descripted only within the patients ever exposed to these treatments.

^b^ The median (IQR) was not presented due to the insufficient number of cases.

**Table 2.** Baseline biochemical indicators of patients with MG and LTBI receiving immunosuppressive therapy.

Variables	Total (*n* = 78)	With a history of immunosuppressive therapy (*n* = 41)	Without a history of immunosuppressive therapy (*n* = 37)	*p*-value
White blood cell count (×10^9^/L), median (IQR)	7.0 (5.7–10.1)	8.0 (6.2–11.0)	6.2 (5.0–7.6)	**.006**
Red blood cell count (×10^12^/L), median (IQR)	4.50 (4.17–4.79)	4.57 (4.18–4.88)	4.46 (4.17–4.73)	.315
Hemoglobin (g/L), median (IQR)	133 (125–144)	135 (128–145)	131 (124–142)	.548
Platelet count (×10^9^/L), median (IQR)	184 (159–233)	177 (151–213)	195 (169–246)	.056
Neutrophil count (×10^9^/L), median (IQR)	4.7 (3.4–7.0)	5.7 (3.6–9.2)	3.9 (3.0–5.4)	**.002**
Lymphocyte count (×10^9^/L), median (IQR)	1.5 (1.1–2.1)	1.3 (0.9–2.1)	1.7 (1.3–2.1)	.066
Monocyte count (×10^9^/L), median (IQR)	0.4 (0.3–0.6)	0.5 (0.3–0.7)	0.4 (0.3–0.6)	.631
Total protein (g/L), median (IQR)	64.6 (59.8–70.2)	62.8 (58.6–70.4)	64.9 (63.1–69.7)	.195
Albumin (g/L), median (IQR)	38.9 (36.3–42.0)	38.8 (35.9–42.6)	38.9 (37.3–41.7)	.841
ALT (U/L), median (IQR)	19.8 (15.4–25.9)	19.8 (15.1–25.6)	19.7 (14.6–29.9)	.806
AST (U/L), median (IQR)	22.9 (17.9–27.4)	21.2 (16.7–25.7)	24.5 (19.1–27.9)	.072
TBIL (μmol/L), median (IQR)	10.4 (7.2–15.9)	10.4 (7.1–16.4)	10.0 (7.5–15.4)	.756
DBIL (μmol/L), median (IQR)	4.4 (3.5–6.3)	4.9 (3.5–6.7)	4.3 (3.3–6.1)	.707
BUN (mmol/L), median (IQR)	4.39 (3.53–6.09)	4.96 (3.83–6.53)	3.80 (3.35–5.04)	**.015**
Creatinine (μmol/L), median (IQR)	73.1 (64.0–81.5)	73.9 (64.0–84.9)	70.0 (64.0–80.0)	.426
Uric acid (μmol/L), median (IQR)	290.5 (243.3–348.2)	283.9 (243.4–347.4)	304.8 (242.0–348.7)	.403
Fasting blood glucose (mmol/L), median (IQR)	4.90 (4.54–5.88)	4.83 (4.46–6.20)	4.90 (4.60–5.79)	.976
Lipid profile	*n* = 72	*n* = 37	*n* = 35	
TC (mmol/L), median (IQR)	4.70 (4.22–5.54)	4.57 (3.81–5.60)	4.77 (4.30–5.54)	.693
TG (mmol/L), median (IQR)	1.48 (1.06–2.20)	1.45 (0.94–2.27)	1.52 (1.08–2.10)	.727
HDL-C (mmol/L), median (IQR)	1.24 (1.07–1.54)	1.24 (1.08–1.54)	1.24 (1.06–1.54)	.861
LDL-C (mmol/L), median (IQR)	2.86 (2.25–3.44)	2.85 (2.21–3.33)	2.89 (2.22–3.56)	.897
K^+^ (mmol/L), median (IQR)	3.80 (3.58–4.02)	3.72 (3.52–3.98)	3.87 (3.67–4.08)	.182
Na^+^ (mmol/L), median (IQR)	142.7 (141.5–143.8)	143.4 (141.8–145.0)	142.2 (141.2–143.3)	**.027**
Cl^−^ (mmol/L), median (IQR)	105.0 (103.1–107.1)	105.6 (103.3–108.0)	104.7 (102.8–106.1)	.108
Ca^2+^ (mmol/L), median (IQR)	2.27 (2.20–2.34)	2.24 (2.17–2.36)	2.29 (2.23–2.33)	.266
Complements and immunoglobulins profiles	*n* = 73	*n* = 38	*n* = 35	
C4 (mg/L), median (IQR)	187 (152–232)	181 (137–225)	198 (162–237)	.099
C3 (mg/L), median (IQR)	796 (692–928)	775 (653–906)	823 (721–947)	.255
IgG (g/L), median (IQR)	11.5 (9.6–13.9)	10.5 (8.9–13.1)	12.9 (10.2–14.5)	**.013**
IgA (mg/L), median (IQR)	1850 (1390–2470)	1640 (1248–2200)	2020 (1550–2720)	**.023**
IgM (mg/L), median (IQR)	1080 (733–1585)	983 (723–1553)	1130 (854–1610)	.675
Elevated ESR, *n* (%)	14 (22.2%) [*n* = 63]	4 (11.4%) [*n* = 35]	10 (35.7%) [*n* = 28]	**.021**
Elevated CRP, *n* (%)	7 (10.4%) [*n* = 67]	6 (17.1%) [*n* = 35]	1 (3.1%) [*n* = 32]	.141
FT3	*n* = 74	*n* = 38	*n* = 36	.671
Elevated, *n* (%)	4 (5.4%)	1 (2.6%)	3 (8.3%)	
Decreased, *n* (%)	2 (2.7%)	1 (2.6%)	1 (2.8%)	
FT4	*n* = 74	*n* = 38	*n* = 36	>.999
Elevated, *n* (%)	4 (5.4%)	2 (5.3%)	2 (5.6%)	
Decreased, *n* (%)	5 (6.8%)	3 (7.9%)	2 (5.6%)	
TSH	*n* = 74	*n* = 38	*n* = 36	.376
Elevated, *n* (%)	5 (6.8%)	3 (7.9%)	2 (5.6%)	
Decreased, n (%)	5 (6.8%)	1 (2.6%)	4 (11.1%)	
Elevated TGA, *n* (%)	8 (11.1%) [*n* = 72]	4 (10.8%) [*n* = 37]	4 (11.4%) [*n* = 35]	>.999
Elevated TPOA, *n* (%)	19 (26.4%) [*n* = 72]	5 (13.5%) [*n* = 37]	14 (40%) [*n* = 35]	**.011**
Elevated TRAb, *n* (%)	6 (20.0%) [*n* = 30]	2 (14.3%) [*n* = 14]	4 (25.0%) [*n* = 16]	.784
ANA positivity, *n* (%)	11 (17.7%) [*n* = 62]	6 (19.4%) [*n* = 31]	5 (16.1%) [*n* = 31]	.740
Anti-dsDNA positivity, *n* (%)	1 (1.6%) [*n* = 62]	0 (0.0%) [*n* = 31]	1 (3.2%) [*n* = 31]	>.999
ANA profile positivity, *n* (%)	12 (20.0%) [*n* = 60]	8 (29.6%) [*n* = 27]	4 (12.1%) [*n* = 33]	.092

ALT: alanine transaminase; ANA: antinuclear antibody; AST: aspartate aminotransferase; BUN: blood urea nitrogen; CRP: C-reactive protein; DBIL: direct bilirubin; dsDNA: double-stranded deoxyribonucleic acid; ESR: erythrocyte sedimentation rate; FT3: free triiodothyronine; FT4: free thyroxine; HDL-C: high-density lipoprotein cholesterol; IQR: interquartile range; LDL-C: low-density lipoprotein cholesterol; TBIL: total bilirubin; TC: total cholesterol; TG: triglyceride; TGA: thyroglobulin autoantibodies; TPOA: thyroid peroxidase autoantibodies; TRAb: thyroid stimulating hormone receptor autoantibodies; TSH: thyroid stimulating hormone. The data were presented as median (IQR) or frequency (percentage). The *p*-value of the comparison with statistical significance (*p* < .05) was shown in bold.

Among the 78 MG patients with LTBI, 10 reported a history of either pulmonary or extrapulmonary TB cured before the enrollment, while 12 exhibited radiographically stable imaging features for TB on pulmonary computed tomography (CT) scans. These patients may experience previous infections of MTB yet were not in active states at the timepoint of enrollment, which in combination included 15 cases. Nevertheless, no discrepancy in the demographic or clinical characteristics including previous exposure to ISTs was observed between MG patients with and without a history of TB or pulmonary TB lesions (Table 1). In this regard, it seemed insufficient to establish an association between the occurrence of previous TB and the application of ISTs.

### Outcomes of MG patients with LTBI receiving ISTs

**Figure 3.**
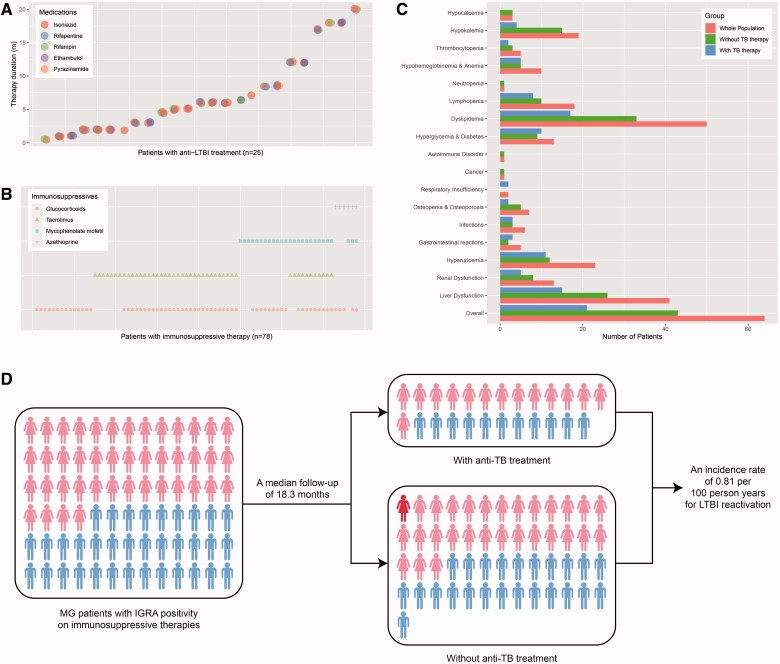
The medications (A,B) and outcomes (C,D) of MG patients with LTBI on immunosuppressive therapies during the follow-up. (A) The type and duration of anti-LTBI treatment in 25 patients. Each cluster of jittered circles represents the medications ever used by a single patient, where their vertical coordinates demonstrate the overall duration of anti-LTBI treatment. (B) The type of immunosuppressive therapies during the whole course of follow-up in 78 patients. Corresponding icons laid lengthwise show all immunosuppressives that were ever taken by a single patient. (C) The number of cases for observed adverse events in the whole populations and subgroups with or without anti-LTBI treatments. (D) The diagram of the conversion from LTBI to ATB in MG patients complicating LTBI and receiving immunosuppressive therapies. The light red and light blue represent females and males, while red indicates the female patient developing LTBI reactivation. IGRA: interferon-gamma release assay; LTBI: latent tuberculosis infection; MG: myasthenia gravis; TB: tuberculosis.

The 78 patients were followed up for a median duration of 18.3 (8.5–24.0) months. During the period, 25 (32.1%) initiated anti-LTBI therapy, with a median treatment course of 6.0 (2.0–10.3) months. All of them were prescribed with isoniazid alone or combined with rifapentine or rifampin, ethambutol, and/or pyrazinamide (Figure 3A). As for ISTs, 83.3%, 59.0%, 33.3% and 7.7% of the patients were ever exposed to GCS, TAC, MMF and AZA over the whole course of follow-up (Figure 3B). Furthermore, two patients used several sessions of rituximab.

At the end of follow-up, Quantitative Myasthenia Gravis (QMG) scores were available in 46 patients, and the median score was 10 (5–17), which was lower than that in the paired ones at the baseline [18 (15–22), *Z* = −4.253, *p* < .001] and suggested effective therapeutic responses for MG. Thirty patients underwent pulmonary CT or X-ray scans, and all of them exhibited either no lesions or radiographically stable signs. Five received microbiologic tests and were all sputum-negative. Twenty-two patients were tested for IGRA and 7 converted to negative results, where only 3 of them received anti-TB therapies. Besides, ESR was found elevated in 3 patients among the 16 screened individuals. The overall incidence of AEs was 82.1% during the follow-up, and the most common was dyslipidemia (64.1%) (Figure 3C). There was no difference in the frequency of AEs between patients with and without TB therapies (*χ*^2^<0.001, *p* > .999).

As for symptoms and signs that may indicate LTBI reactivation, one reported obvious afternoon low-degree fever pattern, excessive sweating, and weight loss (Patient 1), one reported occasional night sweats (also in the daytime) and expectoration (Patient 2), and another reported pronounced afternoon fever for months with intermittent night sweats (Patient 3). Besides, a 45-year-old female patient acutely developed TB at the right mandibular lymph node after 21 months of follow-up and was also suspected for intestinal TB afterwards (Patient 4) (Table 3). When taking various factors into consideration, however, the symptoms of Patient 1 and 2 were more likely to be associated with poorly-controlled hyperthyroidism and side effects of pyridostigmine. For Patient 3, no other evidence for TB was present and the long-lasting fever virtually occurred prior to the initiation of ISTs. Thus, the incidence rate of ATB was discreetly estimated as 0.81 per 100 person years among the observed MG patients with LTBI on ISTs (Figure 3D).

**Table 3.** Detailed characteristics of the patient with LTBI reactivation.

Baseline				
Gender	Female	Age (years old)		45
Disease duration (m)	71.5	Onset type		Early-onset
QMG Score	25	Antibody type		AchR-Ab
MGFA classification nadir	4b	Thymoma		Absence
Time since immunosuppressive therapy (m)	67	Average oral daily GCS (mg)		22.75
Average daily AZA (mg)	0.65	Thymectomy		None
History of myasthenic crisis	Never	Typical TB symptoms		Absence
Pulmonary lesions	Absence	Complications		Hepatitis B
Endpoint				
Follow-up duration (m)	21	QMG score		8
Average daily oral GCS (mg)	20.44	Average daily MMF (g)		0.84
Typical TB symptoms	None reported			
Anti-TB therapy	None (Initiated after the endpoint)			
Adverse events	1. Liver dysfunction; 2. Hyperuricemia; 3. Dyslipidemia; 4. Lymphopenia			
Outcome	1. Tuberculous lymphadenitis of the right mandible; 2. Intestinal TB			

AChR-Ab: acetylcholine receptor antibody; AZA: azathioprine; GCS: glucocorticoids; MGFA: Myasthenia Gravis Foundation of America; MMF: mycophenolate mofetil; QMG: Quantitative Myasthenia Gravis; TB: tuberculosis.

## Discussion

In the clinical management of immune-mediated diseases such as MG, prevention of potential infections, especially for MTB infections in high-burden countries including China [1], represents a matter of great account. On the one hand, the indispensable use of ISTs may put patients at risk of active infections [3,9]. On the other hand, the background of immune-mediated diseases themselves is also likely to contribute to an intrinsic predisposition to either overt or latent infections [10]. Only a few of the currently available studies investigated the relationship between TB and MG. The risk of TB was higher in MG patients, and those with an age > 60 years and using GCS were even riskier for developing TB [11]. The data from a MG center in South Africa revealed that 13 out of 480 MG patients receiving ISTs developed TB during a median follow-up of 3.6 years, and the incidence of TB seemed not to be increased in patients on ISTs though those with a higher maximum dose of prednisone were predisposed to TB [12]. Relative to established TB diseases, LTBI is insidious and generally ignored over the course of ISTs. Scarcely any studies observed the risk of reinfection or reactivation of TB in MG patients with a history of TB disease or LTBI. Merely a case series reported that none of the three MG patients living with human immunodeficiency virus (HIV) and having previous TB developed reactivation during the period of ISTs [13].

Generally, IGRA and tuberculin skin test (TST) are recommended for screening LTBI, whereas IGRA is more specific for being less affected by Bacillus Calmette-Guérin vaccinations and environmental non-tuberculous mycobacteria [14]. As suggested by positive IGRA results followed by excluding indeterminate and potential ATB cases, our observation found that the incidence of LTBI was 35.0% in screened MG patients, which is even higher than IGRA positivity rates ranging from 13% to 20% among the general population in rural China [15]. There lies a possibility that the high IGRA positivity partly resulted from memory immune responses to MTB within individuals with unperceived and underdiagnosed previous infections, particularly in a high-burden country like China. Of note, advanced age and low peripheral lymphocyte counts were highlighted as risk factors for false negative IGRA results [16]. Specifically, receiving ISTs, especially anti-tumor necrosis factor (TNF) agents, may interfere with the performance of IGRA [17]. The indeterminate result of IGRA, which was observed in 1 patient from our cohort, could also mask the trueness of IGRA tests. In this sense, the frequency of positive IGRA in our patients is plausibly still underestimated. Variations among MG individuals indicate that certain subgroup may be easier to contract LTBI. As shown in a population-based study, IGRA positivity rates were higher in males and gradually increased with age [15], which is consistent with findings in our MG cohort.

Notwithstanding the high prevalence of LTBI, no current evidence demonstrated the effects of ISTs on LTBI reactivation among MG sufferers. In our study, only one patient developed extra-pulmonary TB, with the incidence rate of ATB assumed to be 0.81 per 100 person-years, which is comparable to that (0.87 per 100 person years) in individuals tested positive for IGRA from rural China during a 2-year follow-up [18] and even lower than that (1.70 per 100 person years) among all exposed populations with positive IGRA results and followed up for at least 12 months [3]. This implies that it is relatively safe to use standardized ISTs in MG patients, though the low positive predictive value of IGRA could intrinsically lead to the low conversion rate of ATB [19]. Indeed, it has been recognized that anti-TNF agents rather than other biologics such as rituximab are more pronouncedly associated with TB emergence or reactivation [20,21]. Since multifarious immune mechanisms are involved in the host defense against MTB infections with T-cell-mediated responses playing predominant roles [22], targeted therapies for MG may even bring about lower risks of LTBI reactivation. Unfortunately, the low reactivation rate in our cohort renders it inadequate to address the susceptibility to ATB of a specific MG subset, while the identification of male and a history of TB as risk factors for disease reactivation in general populations with LTBI provides a significant reference [18].

Another challenge lies in the tradeoff between the potential of TB reactivation and AEs caused by pre­ventive TB therapies such as hepatotoxicity and neutropenia [2]. Only two of the 480 MG patients received preventive anti-TB treatment when initiating ISTs in the South Africa cohort [12]. In our observation, 25 of the 78 patients were exposed to chemoprophylaxis, varying in regimens and treatment course potentially due to discrepancies among clinicians and poor medication adherence in patients. Of note, reported AEs were seemingly more attributed to the use of ISTs in our observation. There is no consensus on whether preventive anti-TB therapies should be given in MG patients with LTBI and what could be the possible scheme for managing the condition. As stones from other hills, various regimens for preventively treating LTBI in general populations, such as 9 months of daily isoniazid and 3 months of weekly rifapentine plus isoniazid, have been well summarized elsewhere with evidence from randomized controlled trials and recommendations from guidelines [14]. Besides, preventive treatment seemed advantageous against the disease progression among individuals with IGRA positivity [19]. However, in the setting of low reactivation rate like our cohort, the overall benefit may be limited despite the more lasting protective efficacy of preventive therapies [14]. Anyhow, it is more reasonable to merely prescribe TB chemoprophylaxis to patients with LTBI on ISTs instead of to all individuals receiving ISTs, as the risk for ATB was parallel in the two settings yet the former regimen may cause fewer AEs [23]. Like the guidance for rheumatology and dermatology, host-related (e.g. demographic characteristics, underlying comorbidities) and therapy-related (e.g. GCS, conventional non-steroid immunosuppressants, novel biologic agents) factors should be established as well in patients on ISTs for neuroimmunological disorders including MG, thereby assisting in stratifying the risks of LTBI reactivation and guiding the individualized and precise anti-TB treatment [24]. This requires further studies on a greater number of cases with more ISTs applied in clinical practice.

Still, there are various issues that we should pay attention to when managing MG patients with LTBI undergoing ISTs. For instance, drug interactions between rifampicin and TAC may decrease the blood concentration of the latter [25]. Additionally, minor proportions of reversion from positive to negative IGRA results in treated patients and the heterogeneity in concentrations of interferon-γ among populations restrict the values of IGRA in monitoring the efficacy of anti-TB therapies [26]. Even if the negative IGRA tests are present, false-negative errors should also be ruled out especially in the cases of MG patients taking immunosuppressives [17]. Therefore, clinical symptoms and signs indicating ATB should be emphasized during the follow-up. However, symptoms like fatigue and dyspnea could stem from multiple diseases, while hyperhidrosis represents one of the frequent side effects for pyridostigmine [27]. Thus, diagnosis should be prudently made when patients complain about symptoms that resemble those of ATB. Significantly, it is worth noting that extra-pulmonary TB does occur as exemplified by our study. In fact, the predisposition to respiratory and non-respiratory TB was comparable in MG patients [10].

Our work also has some limitations due to all sorts of reasons. First, excluding cases with missing essential records more or less led to selection bias. Second, data reported by patients could introduce recalling bias, whereas the heterogeneity in the tolerance to or awareness of potential symptoms may also affect the determination of ATB. Third, some of the patients in our cohort were clinically diagnosed as LTBI or ATB without microbiological evidence, though it seems practical in real-world settings especially when patients are free from typical symptoms. Lastly, studies involving more biologic agents on larger samples and in multiple centers are required to validate the preliminary results from our observation considering that the present study was retrospectively carried out in a single tertiary hospital.

Overall, notwithstanding the high frequency of LTBI in MG patients from our institution, the use of conventional ISTs for treating MG seems not to aggravate the risk of conversion to ATB. For all MG patients, especially males and those with advanced age, screening for LTBI *via* IGRA is highly suggested prior to ISTs, which not only lowers the false-negative rate but also facilitates the decision on whether preventive anti-TB treatments should be given in the case of IGRA positivity. Concerning MG patients with LTBI on ISTs, it would be better to prescribe prophylactic anti-TB therapies after taking all sorts of host-related and therapy-related risk factors as well as the local prevalence of ATB or LTBI into account. In addition, comprehensive evaluation of TB status is still a must during follow-up when ISTs are applied. Nevertheless, management of MG patients with LTBI on ISTs calls for consensus based on robust evidence to standardize the screening or monitoring schemes of LTBI or ATB and the therapeutic regimens in a risk-stratified manner.

## Data Availability

The authors confirm that the data supporting the findings of this study are available within the article.
